# PCORsearch: A scalable, user-centric platform for self-service cohort discovery and feasibility analysis of PCORnet data

**DOI:** 10.1016/j.compbiolchem.2026.109200

**Published:** 2026-06-22

**Authors:** Jacob Herman, AbdElrahman M. Amer, Soledad Fernández, Neena Thomas, Maciej Pietrzak

**Affiliations:** a Department of Biomedical Informatics, College of Medicine, The Ohio State University, Columbus, OH 43210, USA; b Department of Veterinary Biosciences, College of Veterinary Medicine, The Ohio State University, Columbus, OH 43210, USA; c Center for Biostatistics, The Ohio State University, Columbus, OH 43210, USA

**Keywords:** Cohort discovery, EHR analytics, Research feasibility, Feasibility analysis, Research automation, Deidentified data, PCORnet, Patient-centered outcomes, EHR tools, PCORI

## Abstract

Regulatory and institutional restrictions on access to electronic health records (EHR) result in prolonged delays, often extending several weeks or more, when these records are accessed to assess the feasibility of proposed research. Investigators are restricted from directly querying EHR data servers for feasibility analysis to protect patient privacy, and this responsibility is shifted to independent data analysts. To address this bottleneck, we developed the Patient-Centered Outcomes Research Search Tool (PCORsearch), a web-based set of applications that enables investigators to independently analyze deidentified EHR-derived data while maintaining regulatory compliance. PCORsearch allows users to interactively explore medical terminology codes and data definitions from the PCORnet Common Data Model (CDM) to construct custom cohorts and retrieve feasibility counts. Cohorts are defined using a custom grammar tailored for querying PCORnet CDM data, with attention to usability and security. In addition to cohort discovery, the platform supports the generation of summary statistics and visualizations to further characterize the identified cohorts. Cohorts can be saved and shared between users to aid in collaborative research. The platform ships complete with utility applications to manage and request access to the platform, as well as a companion application where analysts can make use of the web interface and custom grammar to get line-level data access. By streamlining investigator-analyst feasibility assessment workflows, PCORsearch may substantially accelerate early-stage research planning. It enables broader access to large-scale clinical data resources in a modern, secure, compliant manner.

## 1. Introduction

### 1.1. Background

The Patient-Centered Outcomes Research Institute (PCORI) is an independent, non-profit organization established to advance clinical effectiveness research (CER). Since its authorization by congress in 2010, PCORI has become the largest funder of CER in the United States. PCORI identifies research gaps through dialogue with diverse stakeholders including patients, providers, payers, and purchasers and allocates funding based on identified needs (Clancy and Collins, 2025).

One critical gap identified is the limited accessibility to patient data from diverse sources, which often forces medical professionals and researchers to rely solely on internal datasets. Beyond measuring patient outcomes, these datasets are applied to tasks such validating novel algorithms and technology (Liu et al., 2022), heightening the importance of data quality. Such data typically represents limited population, restricting the generalizability of findings to broader patient realities. To address this, PCORI funded the development of the National Patient-Centered Clinical Research Network (PCORnet) in 2013. PCORnet is a highly representative national distributed research network integrates research support from both patients and medical experts to accelerate CER (Collins et al., 2025; Ghildayal et al., 2024).

PCORnet currently includes 85 healthcare institutions organized into nine partner networks, collectively containing data on healthcare encounters from more than 47 million individuals. It enables investigators to pursue research questions that would otherwise be infeasible due to disparate data systems, sparsity, collection, or challenges in collaboration (Collins et al., 2025; Ghildayal et al., 2024). Standardization at this scale is achieved through partners’ adherence to the PCORnet Common Data Model (CDM), which is continually updated to expand its CER capabilities. This data model supports both external data curation and internal feasibility informatics as well as clinical trial recruitment (Collins et al., 2025; Ghildayal et al., 2024).

The Ohio State University (OSU) participates in PCORnet through the PaTH Clinical Research Network (CRN) (Amin et al., 2025). OSU investigators can access line-level limited data set (LDS) and aggregate data from the OSU PCORnet CDM under overarching IRB approval for deidentified viewing (Wolf et al., 2005; Heimer and Petty, 2010). PaTH partners are able to gain similar approval, which is a major benefit of the CRN. Investigators must submit an OSU PCORnet Data Request form, reviewed by the OSU PCORnet Governance Committee. Once approved, an OSU PCORnet data analyst fulfills the request, typically requiring at least one week even for basic cohort size assessments.

Alternatively, investigators may submit requests for feasibility counts directly from electronic health records (EHR) via the Honest Broker Operations Committee (HBOC). However, due to the higher volume and complexity of requests, the HBOC process generally has a longer approval and fulfillment timeline than PCORnet CDM requests. In both pathways, investigators are unable to refine their original queries without submitting additional IRB approvals or amendments.

The current turnaround time for data requests at OSU varies based on queue volume and competing priorities, particularly those requiring CDM support, which are prioritized. Upon receiving a request, the Data Access, Analysis, and Coordination (DAAC) team provides an initial response and schedules a consultation meeting if one has not already occurred. During this meeting, inclusion and exclusion criteria, as well as the specific data elements or aggregate counts requested, are clarified. Once assigned to an analyst, the time required to complete a request depends on its complexity. Simple queries typically require approximately 2–8 h of analyst time, whereas more complex requests may require 20 or more hours. These hours are generally distributed over multiple days to accommodate concurrent responsibilities and ongoing commitments.

Research feasibility assessment, often as simple as determining the size of a cohort of interest (Gatto et al., 2022), is a critical first step that delays early-stage research planning. Investigators may spend weeks waiting for a single SQL query while analysts spend hours sourcing and verifying it, consuming valuable institutional resources (Holmes et al., 2016) and disincentivizing investigators from pursuing their research questions (Zhang et al., 2025). Recognizing this bottleneck, a tool was needed to support rapid feasibility assessments while protected health information (PHI) security.

### 1.2. Platform inception

Several existing tools could theoretically address the need for self-service research feasibility analysis (Schüttler et al., 2021); however, their integration with Ohio State University’s IT infrastructure proved infeasible. This challenge often stemmed from the underlying data models of these platforms. For example, notable systems such as i2b2 (Murphy et al., 2010) (and its derivatives) employ a Star schema data model (Iqbal et al., 1970). In this model, attributes describing different analytical perspectives are organized around a central “fact” table. Various “dimensions” relate directly to the fact table, enabling performance optimizations (Panda, 2025).

In i2b2, the fact table represents an “observation,” a generalized clinical event reflecting a single interaction with a patient. Its *entity-attribute-value (EAV)* design promotes efficient bottom-up data analysis but complicates maintenance and updates over time. To interpret data in an EAV schema, concepts and values within a dimension must be mapped back to their concept identifier, but the tables themselves are agnostic to concepts. This allows for a highly standardized process when specifying concepts within a given domain. To maintain interoperability, new PCORnet concepts would need to be mapped to a new or existing attribute within the Star schema.

Another highly utilized data model, the Observational Medical Outcomes Partnership (OMOP) CDM which is used by OHDSI Atlas, adopts a traditional relational database schema which is much more granular than PCORnet. It is designed like this to promote interoperability at an international scale across varying EHR systems, though data compatibility is achieved in-part by mapping medical terminology codes to *standard concepts*. This means that if some procedure is best represented by a CPT code then all overlapping definitions from ICD, LOINC, etc. will be mapped to CPT. Notably, OMOP is designed for the use case of observational research rather than patient-centered outcomes research; PCOR trials are often interventional and goal-oriented rather than observational and method-oriented.

Compared with these data models, the PCORnet Common Data Model (CDM) adopts a traditional relational database structure similar to OMOP while emphasizing simplicity, flexibility, and portability for existing institutional data (Collins et al., 2025). Each key entity is represented independently: for instance, demographic information is stored in a DEMOGRAPHIC table, while details of healthcare encounters are housed in the ENCOUNTER table. Unlike i2b2, PCORnet does not link concepts through a single observation table (Murphy et al., 2010); instead, it categorizes clinical events across multiple tables by type (e.g., diagnoses, procedures, laboratory results) (Collins et al., 2025). This architecture provides greater flexibility for investigators but entails performance trade-offs for granular analysis.

Due to challenges in establishing robust interoperability between the data models (Hammami et al., 2014), previous attempts to integrate i2b2 with OSU’s IT systems were unsuccessful. The data transformation pipelines created to enable interoperability with i2b2 produced inaccurate results, and correcting them was determined to require substantial development effort and technical maintenance as the PCORnet CDM is updated quarterly. It is certainly possible to transform the data accurately if done carefully enough and proper maintenance times are allocated, but there are benefits to retaining the source format in PCORnet’s case as the CDM is modified and extended regularly, which leaves data transformation infrastructure susceptible to breakage. Additionally, investigators who have leveraged PCORnet data previously would expect to see data following the CDM specification and this could worsen their user experience. Other platforms, such as Daquery from the University of Pittsburgh, were also evaluated but could not be implemented because of Ohio State’s regulations restricting cross-institutional data sharing. In Daquery’s case, it routes all data back to the central Pittsburgh node, which was not approved for OSUMC’s records. EPIC SlicerDicer requires IHIS licensure and active clinical team involvement, limiting its applicability for research purposes (Saini et al., 2021). Finally, while the OHDSI Atlas platform was considered, concerns were raised about its complexity for non-technical users (Shah et al., 2025; Woods et al., 2002). (See Tables 1, 2 for comparison.)

Recognizing these limitations, the OSU-PaTH team identified the need for an accurate, reliable, open-source, and user-friendly platform for interacting with Wexner Medical Center EHR data. More broadly, there is a need for modern and intuitive cohort discovery platforms, as antiquated interfaces and workflows may hinder adoption and reduce investigator efficiency. PCORsearch was developed to address this interdisciplinary gap at the intersection of clinical, computational, and cognitive systems domains by streamlining the entire cohort discovery process (Hu et al., 2024). The platform integrates design elements from existing data analysis tools to streamline EHR analytics, addresses usability challenges across investigators with diverse technical backgrounds, and provides a web application tailored for PCORnet data exploration

### 1.3. Design considerations

PCORsearch is specifically designed to query PCORnet data. The PCORnet CDM is modular and extensible enabling continual expansion to support diverse areas of clinical effectiveness research (Collins et al., 2025). However, this modularity results in data related to a single patient or encounter being distributed across multiple tables and records, as are the medical terminology codes commonly used in EHR queries. Preserving the linkages among these records was a critical requirement to ensure the application’s data integrity. Given the multiple linkage types within the PCORnet CDM, functionality was developed to allow users to specify whether a query should be patient-linked or encounter-linked.

**Fig. 1.**
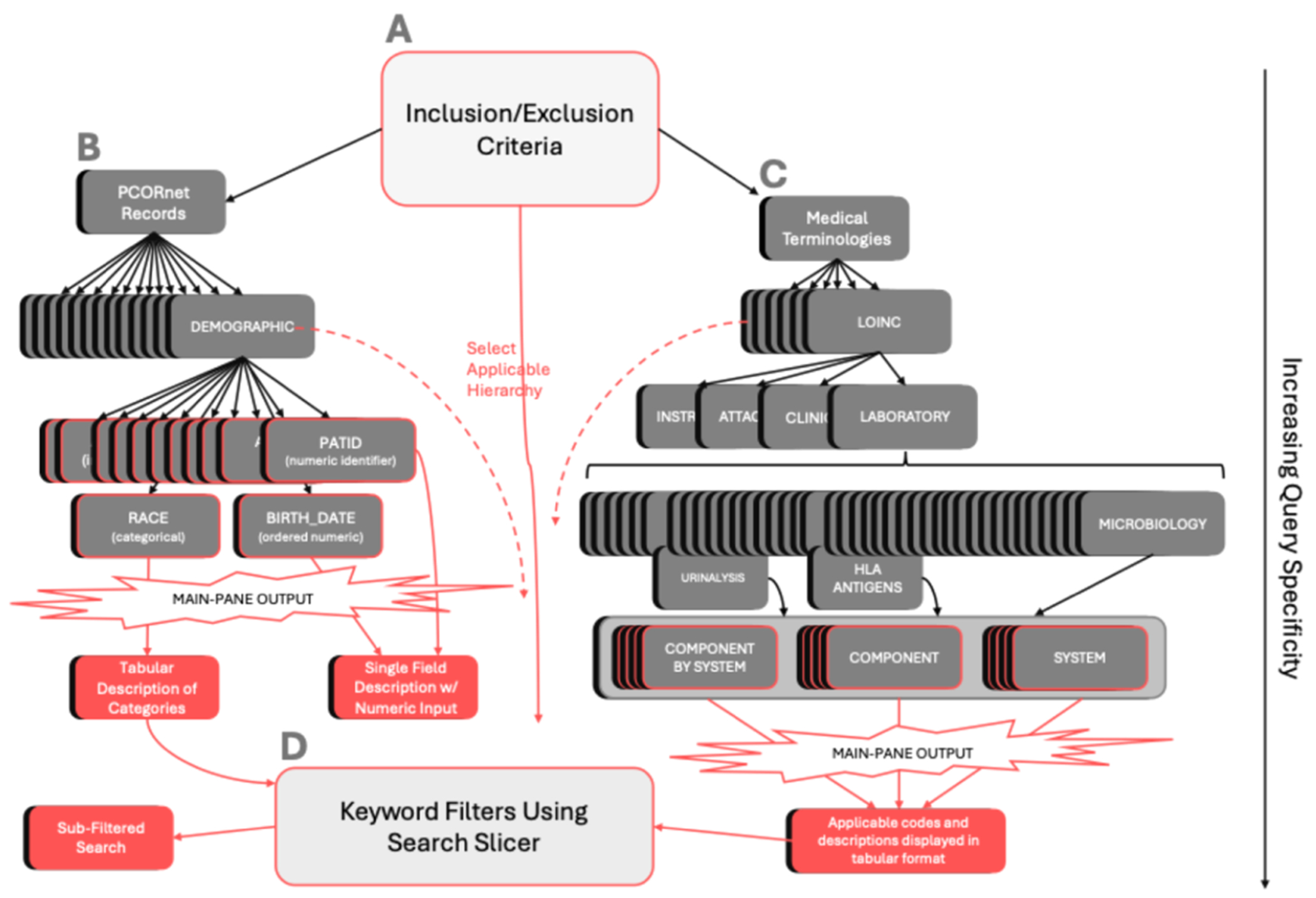
Search Hierarchy and Search Slicer Example – Structure and Outputs for DEMOGRAPHIC and LOINC Searches. **A**. The first step investigators take when using PCORsearch is to formulate inclusion and exclusion criteria. As shown in Fig. 1, this can be accomplished using the search hierarchy and the search slicer tools. **B**. The tab containing PCORnet schema provides a dropdown menu for each table (e.g., DEMOGRAPHIC, ENCOUNTER, DIAGNOSIS), with selectable options for their respective columns (e.g., BIRTH_DATE, RACE). Selecting a column populates the main pane with any available categorical definitions. Interval or ratio columns, such as “BIRTH_DATE,” are handled differently without predefined categories. **C**. The tab containing medical terminology codes includes hierarchies for each code set available in the application (ICD, LOINC, CPT, NDC, RxNorm, and SNOMED). Each hierarchy preserves the original structure of its respective code set. **D**. The search slicer is an adaptive tool designed to accelerate searches for more specifically defined queries. Keyword searches enable users to retrieve initial sets of applicable medical terminology codes (after specifying a code set) or to filter codes already displayed in the main panel.

**Fig. 2.**
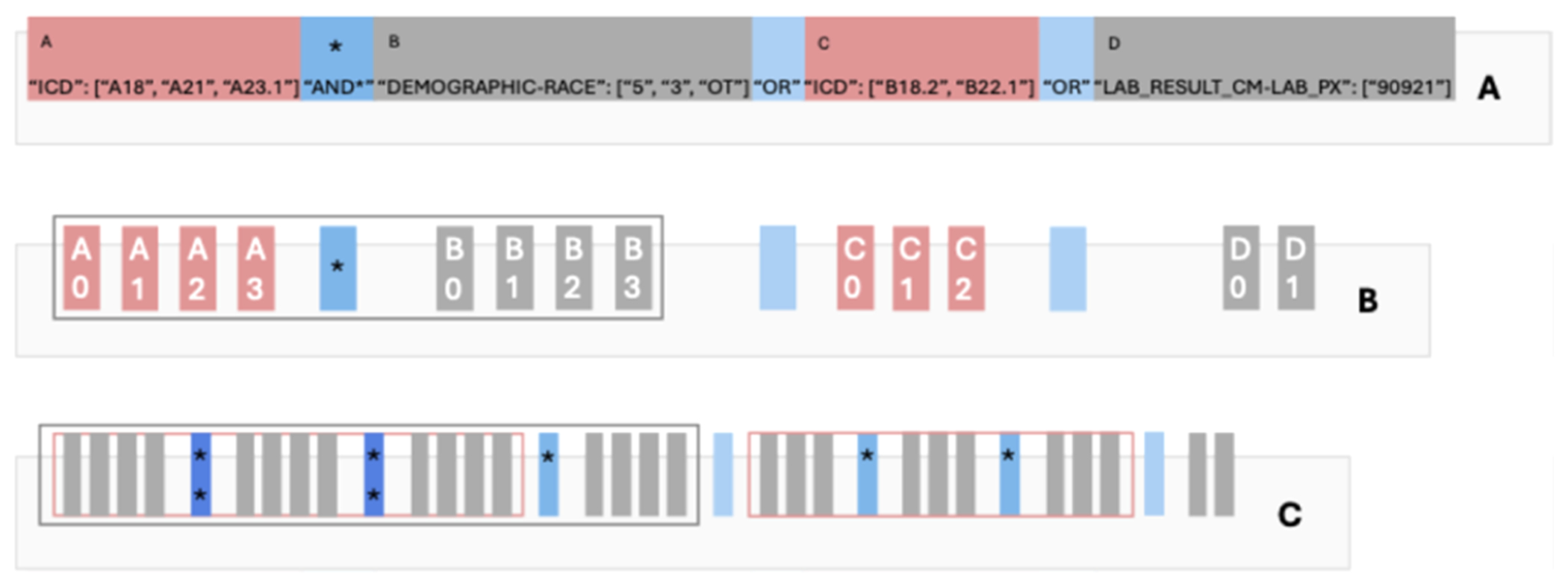
Example of PCORsearch Query Syntax and Overview of Client-to-SQL Request Parsing. **A**. A key feature of PCORnet is the provision of blanket IRB approval for deidentified data access, contingent upon infrastructure ensuring patient privacy. To support this at the implementation level, the PCORsearch client exchanges information with the PCORsearch server exclusively through two methods: search requests and results requests. Shown above is an example of a search request, in which a user specifies the patient cohort they wish to analyze. **B**. Upon receipt at the server, the search request is tokenized. Medical terminology codes are mapped back to their corresponding PCORnet CDM columns, and new key-value pairs are generated. Because valid SQL queries require logical comparators between all key-value pairs, the algorithm automatically supplies comparators while preserving the logical order of operations. To ensure robustness, additional “depth” (representing parentheses in the final SQL) is assigned to comparators based on their own depth and that of adjacent comparators. **C**. After tokenization and mapping, the resulting tokens, now querying only PCORnet CDM columns, are parsed into a valid SQL query and submitted to the database. A temporary view of the user-defined patient cohort is created in the database, enabling the investigator to interact with the cohort through the client interface.

Many investigators have not mapped their research questions into applicable medical terminology codes at the time of making a request, and matching this to PCORnet’s relational schema is time-consuming and requires expertise (Sartipi and Dehmoobad, 2008). To address this, PCORsearch supplies investigators with hierarchically organized definitions from key medical terminology systems, including ICD, CPT, RxNorm, and LOINC. This functionality prevents the need for exhaustive manual searches and helps users, especially those new to EHR data, better understand the structure and use of these terminologies. Importantly, investigators can use terminology codes directly as query terms within the app, promoting efficient and human-readable query formation (Figs. 1, 2, Table 4).

Another major design requirement was to support highly customizable and complex queries while remaining accessible to users without programming backgrounds (Woods et al., 2002). This is needed because facilitating engagement, confidence, and autonomy across a broad user base requires minimizing information asymmetry between technical and non-technical users and ultimately serving the same benefits to both parties (Wang et al., 2026). To achieve this, a flexible query grammar was developed (Supplementary Material 1). This grammar enables novice users (those unfamiliar with SQL or the PCORnet CDM) to populate queries entirely through intuitive graphical interfaces (Woods et al., 2002). Simultaneously, it allows more experienced users to construct queries resembling SQL “WHERE” clauses. A primary design goal was to ensure that the grammar was intuitive enough for investigators to construct queries independently after minimal exposure to examples (Woods et al., 2002). Similar to SQL, which was designed with a human-readable syntax to reduce barriers for non-technical users, PCORsearch acknowledges the inherent complexity of EHR data analysis. The platform is designed to balance efficiency and expressivity in cohort definition by leveraging users’ existing familiarity with language while maintaining an intuitive interface (Table 4).

Perhaps most importantly, it was necessary for the platform to securely handle PHI. As the means of collecting, transmitting, and analyzing healthcare data evolve (perhaps most notably with internet-enabled wearable devices), so have the means of its exploitation. Real-time frameworks process data at a massive scale and may opt for decentralized approaches for seamless and unsupervised verification of data integrity (Zhao et al., 2023). While PCORsearch does not stream data in real-time, it does allow for aggregation of EHR data for approved users, meaning it must take measures to prevent leakage of raw data, prevent hijacking of approved user identities, and mitigate any abuse that does occur (Table 3). This is accomplished by isolating interaction with EHR data to transient subprocesses operating with minimal permissions, encrypting transferred data, integrating institutional identity-provider authentication and manual access management protocols, validating user requests through timestamping and session-consistency checks, assigning regulatory personnel to oversee user activity, and maintaining immutable SQL transaction audit logs. Additional security mechanisms are described under Methods – Security Considerations and in the Formal Threat Diagram (Supplementary Material 2) (Table 3).

Finally, recognizing the broader needs of analysts and regulatory personnel within the Honest Broker Operations Committee, a second version of PCORsearch was developed to support full line-level data access in addition to cohort statistics and visualizations. To ensure regulatory compliance surrounding patient data access (Table 6), two supplemental applications were created: one to provide investigators with an interface to request data access, and another to provide regulators with an interface for managing access permissions.

### 1.4. Performance considerations

PCORsearch is currently configured to query Ohio State University’s PCORnet data repository. As of 2026, this dataset contains over 700 million records across 13 relevant PCORsearch tables (updated quarterly), totaling approximately 290 GB within a Microsoft T-SQL database. Delivering interactive-scale analytics at this scale requires substantial computing power and careful query optimization.

Despite the large data volume, the application is intentionally designed to support flexible implementations with minimal infrastructure requirements. The platform’s architecture emphasizes delaying and isolating heavy computation wherever possible. To achieve this, the computational burden is shifted onto a single lightweight, modular, linear script which can be stored on communal high-performance computing (HPC) resources and invoked only when needed (Li et al., 2023) (Fig. 4). This approach reduces the need for permanently allocated infrastructure and enables developers to adapt the system to environments with limited internal computing resources.

To further optimize performance and reduce hosting costs, PCORsearch dynamically creates views of user-defined cohorts within the database. These temporary views allow the system to efficiently retrieve only the relevant subset of the database for subsequent computations, minimizing the need to scan the full dataset for each query (Kharade et al., 2020). The use of views also means that negligible memory overhead is incurred (only a minimal metadata overhead) in creating user cohorts, though this comes with a performance trade-off. Materialized views may be desired to increase responsiveness, though protocols and infrastructures to manage and prune the stored views would be needed. Finally, during cohort generation, PCORsearch saves memory by using set operations rather than traditional joins (Chaudhuri, 1998). This strategy has a secondary benefit of reducing the likelihood of resource-intensive queries that could potentially cause out-of-memory errors (Table 3).

Together, these design strategies ensure that PCORsearch can provide rapid, scalable analytics while remaining adaptable to a variety of institutional infrastructures and budget constraints.

## 2. Results

### 2.1. Platform description - search

**Fig. 3.**
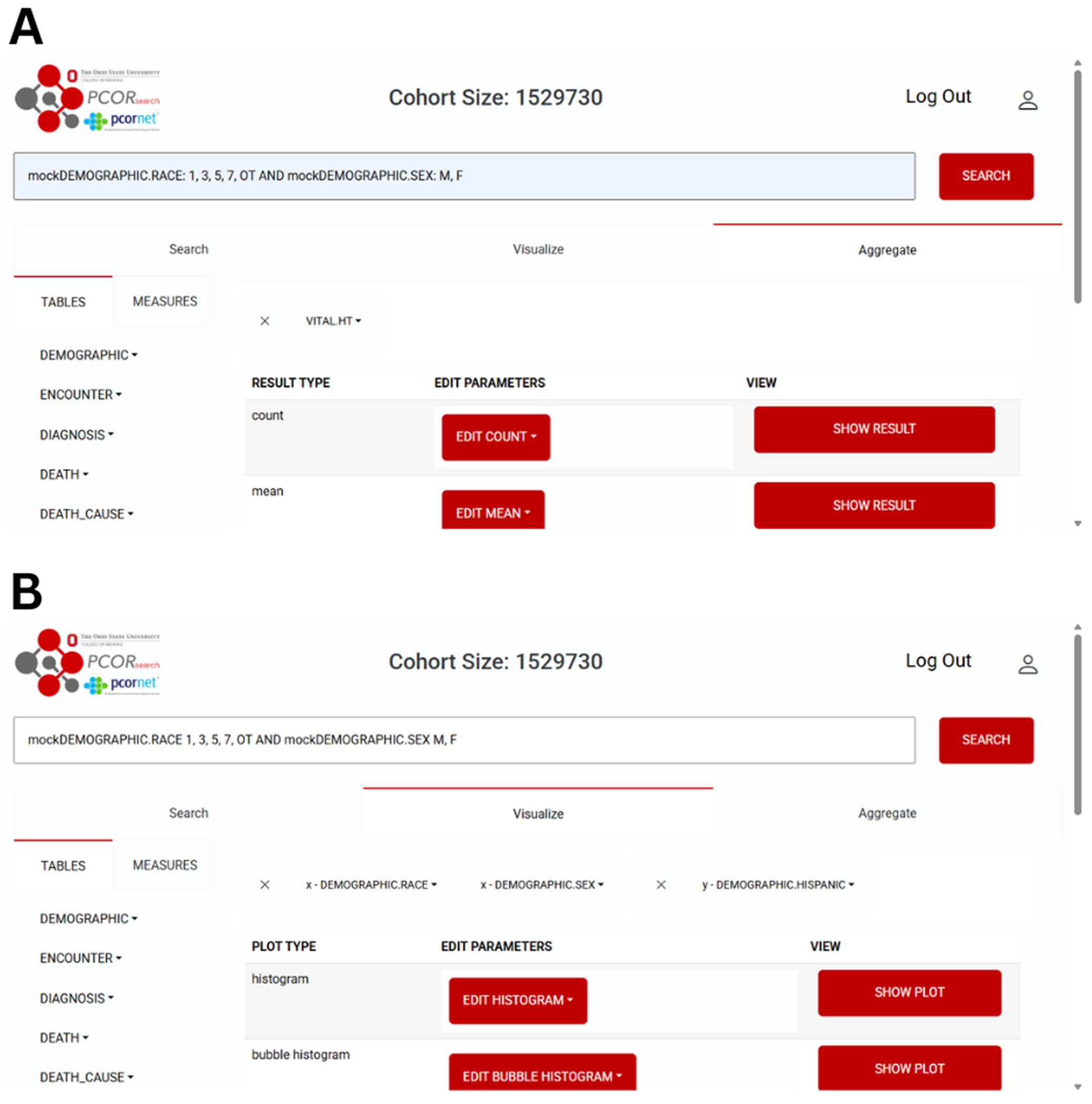
PCORsearch Client Display – Visualize and Aggregate Interfaces. **A**. The Visualize tab provides options for generating a variety of plots. Measures of interest are added to the x-axis and y-axis using the tabs located on the left. The “RECORDS” tab contains PCORnet CDM schema elements, while the “MEASURES” tab holds the user’s query terms from the initial search, with medical terminology codes already parsed into individual PCORnet CDM columns. Plot parameters such as time-series filters, categorical filters, stratification variables, and formatting options can be specified using the “EDIT” dropdown menus. **B**. The Aggregate tab provides options for generating summary statistics. Unlike the Visualize tab, it features a single aggregation axis populated with selected measures. The “RECORDS” and “MEASURES” tabs are available on the left for selecting input measures, and similar parameters to those in the Visualize tab can be specified to customize aggregations.

**Fig. 4.**
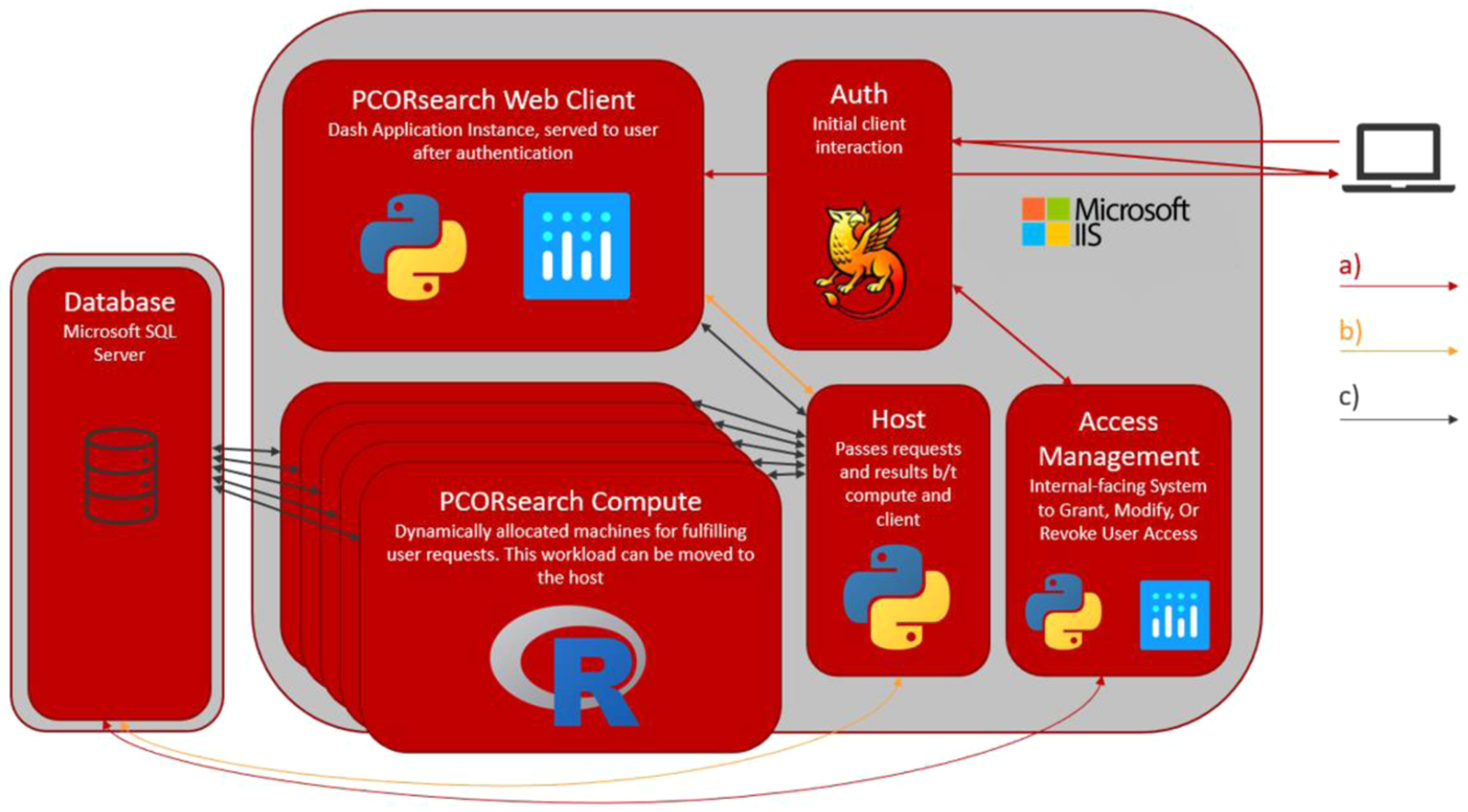
PCORsearch System Architecture Overview. PCORsearch users interact exclusively with the PCORsearch Web Client while the underlying infrastructure manages authentication, query submission, and results retrieval. This abstraction is enabled by standardizing the interfaces through which users access EHR-derived data. Sequentially, the user workflow proceeds as follows: **A**. The user reaches the authentication portal and requests access by submitting a whitelist request to a system administrator. **B**. Once authorized, the user can construct a query within the PCORsearch Web Client, formally submitted as a search request. **C**. The user then selects relevant variables from their identified cohort and submits a results request, the second and final type of request available through the client. The host receives and parses this request, generates a shell script for the compute engine specifying the SQL operations and output instructions, and sends it to the results engine. After processing, the statically encoded result is returned to the host and subsequently delivered back to the user via the client interface. A full tech stack is described in Supplementary Material 3.

**Fig. 5.**
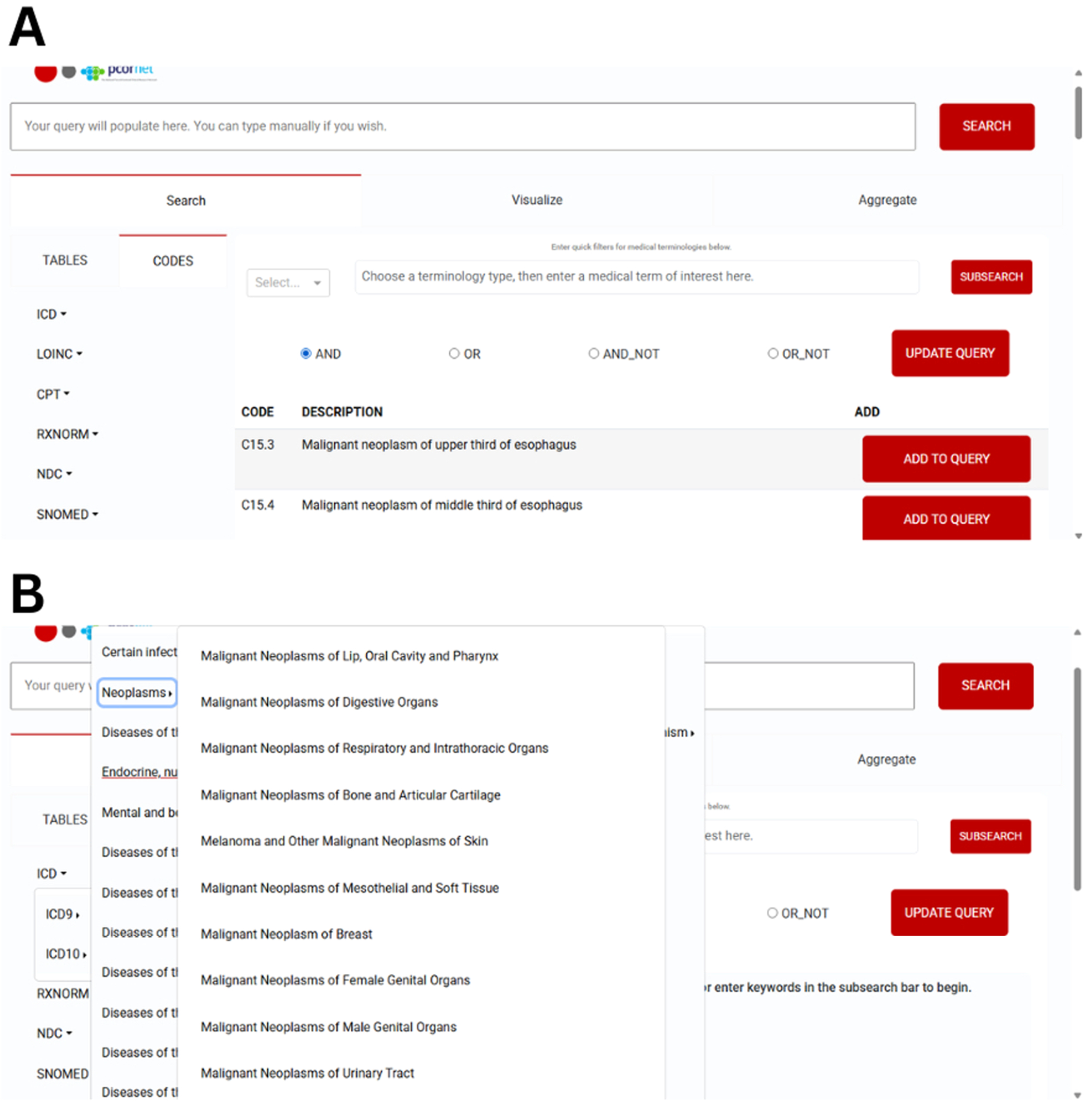
PCORsearch Client Display – Search Interface. **A**. The user is operating within the Search tab to define a cohort of interest. In this example, they are identifying relevant ICD codes under the domain “Malignant Neoplasms of Digestive Organs.” Users can apply additional keyword filters using the search slicer located above the tabular code display; for instance, typing “esophagus” or “pancreas” filters the list to codes containing the specified terms. **B**. The search hierarchy path used to populate the main panel is displayed. PCORsearch supports six terminology code hierarchies: ICD, LOINC, CPT, NDC, RxNorm, and SNOMED. In this example, the user navigated through the ICD hierarchy, selecting ICD-10, Neoplasms, Malignant Neoplasms of Digestive Organs, with the corresponding bottom-level concept automatically populating the code table.

The PCORsearch web client is organized into separate tabs that allow investigators to search for patient cohorts, then visualize and aggregate their results (Figs. 3–5). Analysts can also view line-level data within their version of the application. The initial step in this process is defining a structured SQL query representing the cohort of interest.

The search tab facilitates efficient query formation by centralizing relevant data definitions. Investigators can map their research questions to the PCORnet schema or work independently of it, and construct queries using familiar, intuitive tools that automatically generate query terms (Figs. 1, 2, 5).

Cohort discovery within PCORsearch is performed using two main tools: the search hierarchy and the search slicer. These tools enable investigators to find relevant medical codes and corresponding PCORnet schema components in a flexible, user-friendly manner (Fig. 1).

The search hierarchy allows users to locate appropriate codes even when research questions are not rigidly defined. Six standard terminologies are included: ICD, CPT, LOINC, NDC, RxNorm, and SNOMED. Selecting any bottom-level concept in the search hierarchy displays the applicable codes in the main search pane for inclusion in a query (Fig. 1). Here, a bottom-level concept refers to any grouping of terminology codes that cannot be further subdivided under the same hierarchy type.

In contrast, some terminologies, such as NDC, are not represented as multi-level expandable hierarchies. Instead, they are offered as filterable sets based on relevant properties, with automatic suggestions provided to streamline the search process. This design choice emulates widely adopted tools investigators may already use, promoting a lightweight and familiar user experience.

The search slicer provides an input box for keyword-based searching. Investigators can specify a medical terminology code set using a dropdown menu and then apply keyword filters to refine the displayed codes. It enables investigators to avoid navigating complex hierarchies when a relevant concept is already known. It is also useful for rapidly filtering large lists of returned codes (e.g., filtering more than fifty codes by keyword).

After selecting inclusion or exclusion criteria, users add them to the main query pane by clicking “ADD QUERY” on the desired terms. Clicking the button again removes the term. Users then specify whether the selected terms are inclusion or exclusion criteria and configure logical relationships using comparators (AND, OR, AND NOT, OR NOT) adjacent to the “UPDATE QUERY” button. Pressing “ADD TO QUERY” automatically populates the main query bar. Alternatively, users may manually type their queries.

Inclusion and exclusion criteria are represented internally as key-value pairs, where the key denotes the vocabulary source and the value lists the selected codes (Fig. 1). Comparators are applied between these pairs to build complete queries, supporting button-based query construction even for novice users (Fig. 5).

As mentioned previously, medical terminology codes are automatically mapped back to PCORnet columns during query parsing in a process referred to as “flattening” for convenience. To maintain logical grouping during parsing, parentheses are added around flattened terms. The query parser assigns a “depth” value (the number of parentheses needed) to comparators, ensuring that logical relationships are correctly preserved. Advanced users can further control grouping manually by adding parentheses to their query in the same way one would control the order of operations of a SQL query or math equation. Another advanced feature is the ability to modify the scope of a single comparison within a given query. Cohorts are represented by a list of PATIDs or ENCOUNTERIDs and are formed by selecting PATIDs meeting inclusion criteria. Adding “AND ENCOUNTER WITH (…)” or “PATIENT WITH (…)” before a set of terms will allow for included rows to have a match with that specific identifier, allowing for increased temporal specificity over a cohort’s medical history.

Another feature of the PCORsearch grammar is support for intuitive date, range, and Linux shell-like wildcard filtering (Supplementary Material 1). Any column that can be parsed as an integer (including dates) can be operated on by using “>”, “<”, and “TO” between a single or two values, respectively.

Overall, the PCORsearch query syntax is flexible. Users may omit braces, colons, commas, and quotation marks if desired. A typical valid query follows the general pattern:

(VOCABULARY1 TERM1.1 … TERM1.N COMPARATOR1 VOCABULARY2 TERM2.1 … TERM2.P COMPARATOR2 VOCABULARY3 > TERM3.1) COMPARATOR3 XXXX.XXXX_DATE YYYY-MM-DD TO YYYY-MM-DD

This structure ensures that both novice and advanced users can efficiently formulate complex cohort discovery queries within the platform. An overview of the query parsing algorithm is described below.

Before being parsed into SQL, user queries are broken into tokens across spaces and then flattened. During flattening the tokens are scanned for keywords like ICD, and if found, the values associated with that keyword are replicated into every relevant PCORnet column and wrapped in parentheses. Next, the tokens are assessed for modified patient/encounter scope and wildcard terms. These span multiple tokens in the initial space-wise parsing method, so they are merged into a single token. After this, the tokens are separated into individual terms (using a custom *Term* object, keys and values with added metadata for scope, parentheses, etc.) then checked for malformed inputs. This includes scanning for disallowed query terms, mismatching parentheses, invalid comparator terms, and more. Errors found here are reported back to the user on the web client to assist in cohort discovery and logged internally for administrative review. If no errors are reported, the term list is directly built into a SQL string by iterating through the Term list and performing logical comparisons.

PCORsearch’s combination of robust error detection and performance optimizations affords increased flexibility to users, allowing investigators to freely utilize the cohort discovery syntax within their approved study criteria. Performance is reported in greater detail below; however, execution time scales linearly with both the number of records scanned and cohort depth due to the use of set operations rather than joins. In internal testing, cohort definition times remained under two minutes across evaluated workloads, supporting the platform’s ability to handle the anticipated request volume. Performance degradation primarily occurs under conditions of elevated SQL server workload, emphasizing the importance of sufficient worker allocation and memory availability for concurrent transactions.

### 2.2. Platform description – visualize and aggregate

After executing a query, investigators can utilize the Visualize and Aggregate tabs within the PCORsearch web client. Each tab provides a side pane listing PCORnet columns that can be selected as measures of interest. Additionally, any measures used during the initial query are automatically populated in the side pane under the “MEASURES” section (Fig. 3).

In the Visualize tab, investigators can assign an arbitrary number of measures to the visualization’s x-axis and y-axis (Murray, 2013), populating the main panel. Available visualization types are then displayed dynamically based on the selected data types and axis assignments. The Aggregate tab follows a similar design; however, measures populate a single aggregation axis rather than two (Fig. 3).

Each visualization or aggregation result provides parameter options accessible through the main interface. For instance, the Color parameter allows users to customize plots by either specifying a color name or hexadecimal code or by specifying a column to stratify results. The Time Series parameter automatically detects encounter dates associated with selected measures, enabling investigators to filter results over specified time intervals or partition results across the time axis. All results are rendered as static images or text outputs, which investigators can readily save or copy.

**Fig. 6.**
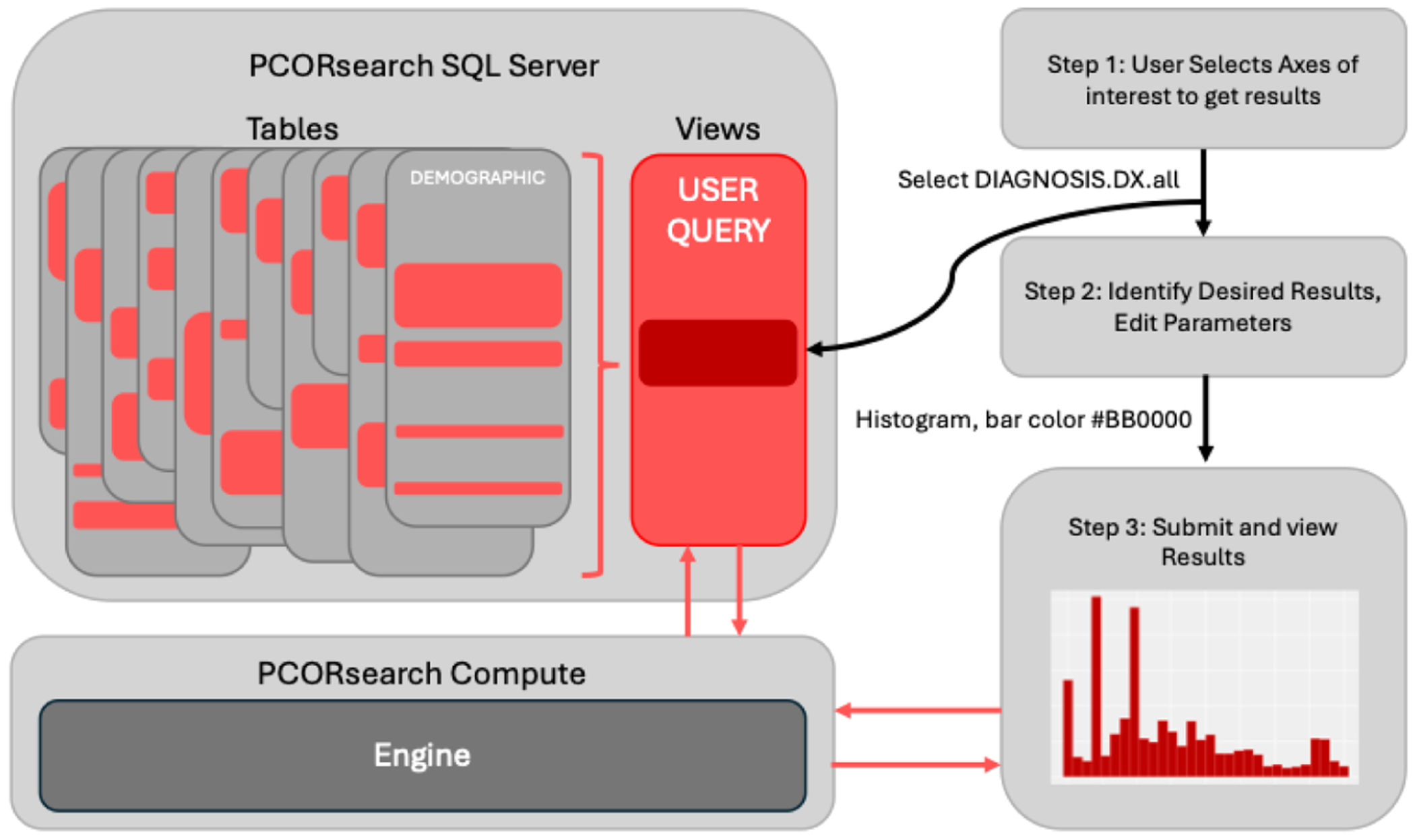
Example of a Results Request Process in PCORsearch. The second and final method by which the PCORsearch client interfaces with the server is through a results request. To optimize efficiency and maintain security (Kharade et al., 2020), most computational processing is abstracted away from the client. The PCORsearch client automatically loads available parameters for a selected result, which users can specify through the “EDIT” dropdown menus. These parameters, along with the request type, are sent to the PCORsearch server, which generates a shell script based on the request. The script is then transmitted to the PCORsearch compute engine and contains instructions for the SQL query and the result to be generated. Upon completion, the compute engine returns a statically encoded result to the host, which subsequently delivers it back to the client interface.

Following result request submission the request is parsed and routed similarly to search requests, but the two differ in their backend handling. Unlike search requests, which are translated then sent directly to the SQL server, results requests are dispatched by invoking an isolated subprocess to promote security (Jiang et al., 2026). The results generation script reads request headers, extracts the relevant measures, and generates the requested visualizations or aggregations as static outputs (Fig. 6). The results generated are then routed back through the host and delivered to the web client. Importantly, the GitHub release hosts the result generation script on the same machine as the Flask application; deployments that place the script on a separate machine would need to replace the subprocess invocation with an HTTP request.

PCORsearch’s results engine is designed to accommodate queries ranging across multiple orders of magnitude in size. To maintain system responsiveness and security, the results engine operates independently from the core application processes in a de-elevated shell environment. Processes within the results engine are initiated dynamically upon user request, ensuring that resources are only consumed when necessary (Fig. 5). The result generation algorithm is described at a high level below.

First, the host receives the plot type, columns to be visualized, and additional parameters. The columns to be visualized are used to create relevant SQL queries in this form: SELECT column_of_interest FROM table_of_interest WHERE table_of_interest.cohort_joinkey IN SELECT cohort_joinkey FROM USER_QUERY_<name> . The plot type, generated queries, and other parameters are passed into the result generation script as arguments. The R script validates the argument schema, executes the supplied SQL queries to gather the correct data, opens a tempfile for output, then calls the correct plot generator function based on the plot type argument. All the plot generator functions apply plot-specific data transformations before calling the plotting/statistical function. The ggplot2 package is used for plotting functions while base R is used for statistics. The results are encoded and written to the temporary file. The host reads this temporary file, deletes it, and broadcasts the results back to the client.

### 2.3. Performance benchmarking

PCORsearch is performant enough to handle the data volume and request load at OSU on a shared SQL server (24 GB RAM total, holding 290 GB EHR records) and shared application server (32 GB RAM total). Benchmarks for single-query latency and application throughput analysis conducted on the Flask host are described in Table 5.

Low complexity and high complexity queries were used for latency benchmarks. A low complexity query is defined here as a query that has two key-value pairs at most. A high complexity query is defined as containing at least five key-value pairs and reaching a maximum depth of at least 2. Another consideration is that the PCORnet table LAB_RESULT_CM is much larger than all the other tables used in PCORsearch, and queries against LAB_RESULT_CM will slow down execution. The representative benchmark queries are provided as supplemental data.

Small-scale throughput tests conducted with institutional regulators suggested that the platform is capable of handling several concurrent users and requests; however, identity-verification and manual-accessmanagement requirements prevented reliable automated throughput benchmarking using tools such as Locust. As such, load analysis will be conducted upon release.

Preliminary feedback from institutional analysts suggested that PCORsearch could reduce the iterative investigator–analyst communication typically required for cohort refinement and feasibility assessment and provide investigators with greater autonomy in independently exploring cohort definitions and available data elements. Traditional workflows were estimated to require approximately 2–4 weeks of clarification and refinement prior to feasibility querying, followed by an additional 1–2 days for cohort counts, whereas self-service cohort exploration through PCORsearch could streamline this process and reduce delays associated with repeated feasibility-query refinement. The analyst and end-user testing was also an opportunity to improve the platform. Some examples include: 1) adding more detail to the instructions to the user interface; these tests were conducted by users who were unfamiliar with the platform, and they ended up needing more guidance on how to specify data for result generation. 2) For similar reasons, the cohort discovery grammar was made to be more flexible; users did not want to put whitespace between every operator in the grammar, so the parser was relaxed to break across recognized operators as well as whitespace. 3) For the auxiliary apps, more robust input sanitization was added to the access request page, as people were entering OSUMC credentials that could not be validated with Shibboleth SP. 4) A medical terminology code parser was added to the access management application so that regulators could easily understand the queries being made. Last, every app received minor formatting adjustments to improve readability.

### 2.4. Infrastructure setup for production environments

PCORsearch is designed to support rapid deployment, but implementors must still adhere to regulatory guidelines and obtain the permissions necessary for launch. Importantly, many of these regulatory processes are relevant because PCORsearch is designed to stand as a publicly exposed web application. In other words, teams choosing to download local instances of PCORsearch will avoid many time-consuming regulatory delays if they can connect their instances to an existing SQL server (Table 6). This may be useful to test out the platform before committing to a full deployment.

There are two types of regulatory processes that need to be followed in implementing PCORsearch: processes to mitigate/assess the potential for a breach of PHI and processes to create a publicly facing web app affiliated with a university research hospital. At OSUMC, several significant IT services were identified, as summarized in Table 6.

To create a functional locally hosted version of PCORsearch, only the source code, an existing PCORnet SQL server, and the necessary regulatory clearances are required. In this configuration, users obtain access through the locally hosted request application, where study criteria are documented, data use agreements are signed, and identity is verified. While some form of identity verification is always required, institutions may need to modify the source code to replace Shibboleth SP with an alternative identity provider. Identity verification is performed when requests are submitted to the host, users navigate between pages, or SQL queries are executed. Relevant environment variables and more must also be set, so these details are specified within documentation on the platform’s GitHub repository. The primary implementation challenge in this deployment model is coordination with institutional IT services to establish identity-provider integration, whereas the required code modifications themselves would likely require only limited development effort. Modifying the identity verification code to utilize traceable shared secrets (physically distributed during the meeting with a regulator to request access) would be a reasonable way to avoid this bottleneck but would be less secure and might be subject to additional institutional constraints.

The regulatory processes listed above can take several months to complete; therefore, a full implementation of PCORsearch requires thorough planning. In contrast, a locally hosted implementation can be deployed much more rapidly, suggesting that regulatory and institutional requirements surrounding EHR data, rather than the platform itself, represent the primary barrier to deployment.

### 2.5. Platform limitations

PCORsearch is undergoing continuous improvements and updates to enhance its capabilities and improve the user experience. Many of these improvements are made to target the primary limitations of the platform, data opacity and user expressivity. Regarding data opacity: since PCORsearch only returns aggregate results, it can be difficult to understand what data (lab results, etc.) is held within a cohort. To alleviate this, the aggregation pane allows users to automatically retrieve definitions for medical terminology codes recorded in the EHR. *Count* requests return counts of unique values within a column of data, and there is a button to parse these values (which might look something like “U07.1”) into their relevant codeset (making “U07.1” → “COVID–19”).

Regarding expressivity: users will benefit from defining a cohort in a way that is accurate while writing like concise, plain English. English has more granular comparisons than the binary AND/OR operators supplied in PCORsearch, so more operators are being added to the platform. Examples of these include the wildcard symbol, the “PATIENT/ENCOUNTER WITH” join key scope modifier, and the “> /< /TO” range operators. The next planned addition to the grammar is a “BEFORE/AFTER” statement, as there is currently no simple way to establish self-referentiality between query terms (for instance, if you wanted to find patients who received rhinoplasty after a facial injury).

A third limitation is that PCORsearch currently holds data from a single site while PCORnet is a multi-site CRN. Utilizing data from multiple sites within the CRN would improve the representativeness of the data and enhance the generalizability of investigator results. The requirements for a multi-site implementation of PCORsearch are expanded upon in the discussion.

## 3. Discussion

Existing feasibility informatics tools are well-established and serve a variety of user needs. However, common design choices have limited their application in certain contexts (Table 1). Specifically, the extensive feature sets of many existing platforms can be challenging for less experienced users, potentially affecting adoption. In contrast, PCORsearch was developed with a focus on simplicity, prioritizing user efficiency and intuitive design to support the most common use cases, even at a potential cost to flexibility in addressing highly irregular or granular research questions.

Because PCORsearch is integrated with Ohio State University’s IT infrastructure and OSU investigators have blanket IRB approval for access to deidentified PCORnet data, authorized investigators can use the platform autonomously and obtain interactive-scale feasibility results without external assistance, avoiding the multi-week delays typically associated with data requests.

PCORsearch’s relatively small codebase further enhances its accessibility, making it an attractive option for small teams or individuals seeking to develop their own local implementations. Its modular architecture promotes flexible deployment across environments with varying levels of computational infrastructure. Most platform components are portable across institutional deployments. The only non-portable infrastructure is that which is directly tied to the physical machine hosting the app and OSUMC’s IT services, though the same general implementation strategies can be followed in most cases. Specifically, the elements and code snippets which need modification or recreation are the reverse proxy gateway server through IIS, configurations for the institutional identity provider (including the identity. aspx endpoint file, discussed later and in Supplementary Material 2), code for automated email with SMTP, and internal secrets used for authorization.

In summary, PCORsearch minimizes the learning curve for investigators, reduces setup time for developers, and ensures regulatory compliance through blanket IRB approval. It offers a modular feasibility informatics framework that could be adapted for deployment across PCORnet partner institutions.

Several future developments could further improve the usability and applicability of PCORsearch. One potential enhancement is support for heterogeneous data sources. The current implementation queries the full institutional PCORnet repository; however, smaller, investigator-specific data sources could support more lightweight and targeted deployments. Such an approach may reduce computational overhead and improve flexibility, although potentially at the cost of demographic breadth and statistical power, both of which are key advantages of PCORnet-scale analyses.

This highlights a primary limitation of the current implementation: although PCORnet is a multi-site research network, PCORsearch currently operates on a single institutional data source. Investigators pursuing narrowly defined or data-intensive research questions may therefore encounter limited cohort sizes and reduced population diversity compared with a multi-site PCORnet analysis. Expanding PCORsearch to support cross-site cohort discovery could improve both statistical power and cohort representativeness while more fully leveraging the scale of the PCORnet network (Li et al., 2022; Feng et al., 2025).

Because PCORnet is standardized, a cross-site implementation of PCORsearch is technically feasible. However, the primary challenges are regulatory, requiring coordination of multiple IRBs, data use agreements, liability provisions, and state-specific approvals. PCORsearch is already designed with many of the necessary scalability considerations to support large-scale implementations, so most of the technical work would be in ensuring regulatory compliance. Implementing a system with multiple layers of aggregation and privacy protection would likely be the main requirement to prevent leakage of raw data across institutional boundaries. Additionally, cross-site communication channels, reporting standards, and management systems for regulatory personnel would likely also be required for regulatory purposes.

Finally, integration of natural language processing into cohort discovery represents a planned future extension of the platform. It is likely that pretrained frameworks like PubMedBERT (Gu et al., 2021) will be utilized as the backbone for biomedical language understanding. With this feature, users would enter text in the search slicer describing their cohort (potentially pasting in the study plan they wrote to request access). Clinical meaning would be extracted and aligned with the medical ontologies used in cohort discovery to produce multiple vectors representing different terms. Since these study plans are often multifaceted and highly detailed, the framework will almost certainly have to go beyond the naïve approach of creating global feature embeddings for the study descriptions and returning the codeset terms whose embeddings minimize the squared error loss to specifically return every relevant term. Recent advances in deep learning-based named entity recognition (NER) have demonstrated the utility of multi-channel contextual architectures for extracting heterogeneous clinical meaning from text (Wang et al., 2025), supporting the feasibility of this proposed extension. Implementing this would better achieve PCORsearch’s goal of ease of use, as it could allow users to skip navigating through codesets and instead focus on constructing their cohort. PCORsearch’s ease of use is also being regularly improved through additions to the cohort definition syntax, as doing so allows for more precise cohort definition with less effort.

## 4. Methods

### 4.1. PCORsearch data model

Version 17 of the PCORnet Common Data Model (https://pcornet.org/data/common-data-model/) includes 24 patient-related tables. Each table contains a unique line-level identifier and, where applicable, additional identifiers linking records to specific patients or encounters. Of these, 13 tables are exposed to investigators through PCORsearch to support cohort discovery and feasibility analysis (Table 7).

### 4.2. Architecture overview

PCORsearch is designed with efficiency in mind for developers as well as investigators. The platform operates through eight modular “building blocks,” enabling flexible deployment across a range of infrastructures. These components include the client interface, the host, the results script, the authentication service, the access request interface, the access management interface, the landing page, and the SQL server.

A key architectural consideration was to isolate the result generation process from the host. Because investigators have significant freedom in querying the database, resulting cohort sizes can vary across multiple orders of magnitude. Consequently, any hardware handling cohort data must be capable of holding millions of records in memory. To address this without incurring prohibitive hosting costs, PCORsearch minimizes persistent computation by generating results dynamically through a single R script. The script can reside on a dormant machine or optionally, on the host machine itself, and are called only when needed to process user queries (compatible with serverless computing resources) (Li et al., 2023). This architecture allows developers to integrate PCORsearch with job-based high-performance computing (HPC) resources (Ohio Supercomputer Center, 1987), thereby minimizing reliance on extensive internal infrastructure. Additionally, PCORsearch can be operated in environments lacking dedicated servers by deploying the client and host applications on a local machine and routing result requests through HPC resources (subject to compliance with institutional security requirements (Murphy et al., 2011); Wolf et al., 2005).

This isolation also facilitates security as the result generation scripts can be de-elevated to remove all permissions outside of executing the result generation script and reading necessary configuration files for the SQL server.

All users are required to authenticate prior to accessing the platform. In Ohio State University’s implementation, users sign in with their institutional credentials through a Shibboleth Service Provider (Afifi,). Upon initial authentication, users must also be whitelisted by a system administrator to access the client. Users who are not authorized will be directed to a page where they can submit a justification for access approval.

The PCORsearch web client is supported by a host application, which operates minimally by routing data between the client and results engine. The host primarily provides endpoints to interpret and manage user requests.

Ohio State’s implementation houses PCORsearch data on a Microsoft SQL Server instance. As of March 2025, the dataset totals approximately 290 GB. Since user queries can potentially scan the full dataset with multiple complex conditions, it is essential for developers to allocate sufficient resources to the SQL server to ensure acceptable performance levels.

### 4.3. Security considerations

**Fig. 7.**
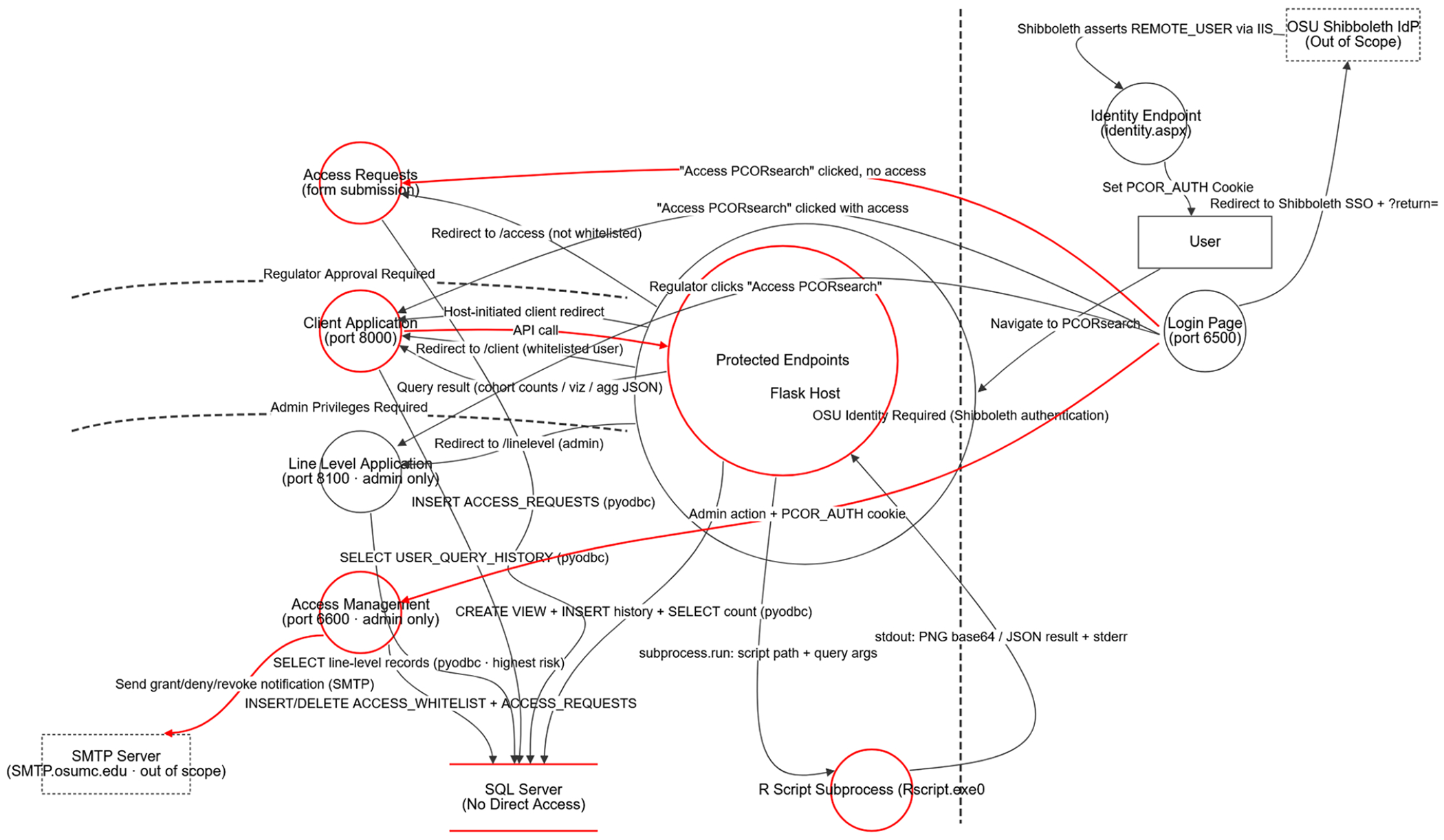
PCORsearch Security at a High Level. The platform consists of three authorization levels: OSU Affiliation, Regulator Approval, and Admin Rights. First, only users with an Ohio State user account (verified with Shibboleth IdP and Duo two-factor authentication) are allowed to make data requests via the Requests webpage. Users without an OSU identity will not be able to navigate past the landing page. On the access request webpage, a user must describe their study’s inclusion/exclusion criteria, accept data use agreements, and give a signature to be reviewed by OSU data regulators. Data regulators must be satisfied in the specificity of the inclusion criteria to award a user access to the web client. After getting access to the client, cohorts a user generates are linked to this study description. This allows regulators to monitor for misuse of resources. Regulators have admin-level access (specified in write-protected configs), giving them the access to apps which manage incoming requests and active users.

PCORsearch incorporates multiple layers of authentication, authorization, data protection, and threat mitigation. The platform has undergone internal security review, and a formal threat analysis based on the STRIDE framework (Johnstone and Michael, 2016) was conducted to identify potential vulnerabilities (Fig. 7, Supplementary Materials). An overview of this analysis and the corresponding security controls is provided below.

At Ohio State, PCORsearch is integrated with the college’s identity provider service, Shibboleth. This platform allows for two-factor authentication of every user, mitigating identity spoofing. One major consideration is that cookies from Shibboleth are validated at an identity endpoint, a necessary step for transfer of credentials between the host’s data endpoints (Fig. 7, Supplementary Material 2). This poses some security risks for identity spoofing, which are mitigated by strictly monitoring for stale cookies and requiring handshakes using internal secrets when data is requested.

The platform does not write data on the server side outside of end-points for access requests and activity logs written to SQL (which are protected against SQL injection and arbitrary execution), leaving little room for tampering attacks there. The most concerning point is the cohort generation step, since this involves interaction with SQL through a custom grammar. Standard input sanitization and whitelist-based validation of referenced tables mitigate most risks associated with query execution.

Repudiation attacks are also easily mitigated, as the main dishonest action taken would be to examine a cohort outside of the study criteria described when requesting access. Regulators monitor PCORsearch users via the access management interface and revoke access (with potential for permanent blacklisting and other disciplinary action following investigation) if a user is generating extraneous cohorts (Fig. 7, Supplementary Material 2). Regulatory abuse is also a possibility, which is mitigated through an immutable SQL Server audit log on all transactions within the database. A reasonable practice is to screen or broadcast these logs often as/to an entire regulatory unit to promote accountability.

Information Disclosure is the key consideration for this app’s claim to security. The app cannot return line-level results to users whatsoever. The data is protected from exposure (both maliciously and by accident) in-part by preventing the application processes from ever interacting with the raw data. To manipulate data and generate results, subprocesses (the result generation script) are invoked with minimal permissions. The subprocess handles fetching the appropriate cohort and linking the cohort to necessary data fields for result generation by itself, and it only responds with aggregate data (Fig. 7, Supplementary Material 2). Additionally, user cohorts are associated only with patient or encounter identifiers rather than complete database tables. There is no method on the client app or host to see raw data apart from looking at a user’s cohort history (which is protected from SQL injection, monitored, and linked to a user’s identity).

PCORsearch at OSU sits behind OSUMC’s firewall and is tied to OSU identity, preventing most attackers from executing a denial-of-service attack. Rate-limiting is implemented in the platform to mitigate this concern further.

Users who gain administrator privileges would be able to access line-level data. For this reason, administrator privileges are stored separately from the SQL server to completely prevent a SQL injection-based elevation attack. The privileges are instead defined in the environment of the application-specific user account. Implementations of PCOR-search sitting on outside of an institutional firewall like that of OSUMC should consider cloud-based secure storage or some other option to safely manage these credentials and other internal secrets.

## Supplementary Material

Supplementary Materials 1

Supplementary Materials 3

Supplementary Materials 2

## Figures and Tables

**Table 1:** Comparison of Existing Feasibility Informatics Platforms, Infrastructure.

Platform	PCORsearch	i2b2	EPIC SlicerDicer	ODHSI ATLAS
Licensure	Open-Source	Open-Source	Proprietary	Open-Source
IRB Approval Required?	**No**. Universities can get **blanket approval** for deidentified access to PCORnet data.	Implementation-Specific and Data-Specific	Yes	Implementation-Specific and Data-Specific
UI Feature Complexity	3 Tabs for cohort definition, visualizations, and statistics	3 Tabs for cohort definition, analysis definition, and analysis within Analysis Container	Point-and-Click Dashboard for cohort filters, summaries, and visualizations	15 Tabs for data source selection, cohort definition, analytics, treatment pathways, study design, etc.
Initial Release	2025	2004	~2016	2014

**Table 2:** Comparison of Existing Feasibility Informatics Platforms, Features.

Platform	PCORsearch	i2b2	EPIC SlicerDicer	ODHSI ATLAS
Medical Terminologies	Yes	Yes	Limited, Uses EPIC vocabularies	Yes
User-Data Interface	**Query-based**. For ease of use, performance, and regulatory reasons, the PCORsearch client is entirely stateless.	Object-based. Users import a data source, then define and modify a cohort within the client.	Static object-based. Users can modify a pre-defined cohort within the client.	Object-based. Users Import a data source, then define and modify a cohort within the client.
Statistics	Yes	Yes	Yes	Yes
Visualization	Yes	Limited, Plugin-Based	Yes, Interactive	Yes

Note: Due to 1) differences in source data models resulting in different strengths and 2) minimal deidentified testing data approved for use outside of PCORsearch, a quantitative analysis of performance across these tools was not conducted. Such an analysis could be considerably biased and therefore ill-posed. However, omission of direct cross-platform benchmarking also introduces limitations in comparative evaluation. Internal performance benchmarking is highly dependent on the underlying computational infrastructure and may vary substantially based on allocated platform and SQL server resources. The production infrastructure for PCORsearch at OSUMC was used for performance benchmarking (Table 5), which we believe to be most representative of a typical environment where it would be hosted.

**Table 3:** Summary of Implementation Objectives in Developing PCORsearch (Witte et al., 2023).

Objective	Design Goals	Obstacles	Results
Security (Kharade et al., 2020)	1) Prevent direct exposure of raw data. 2) Prevent vulnerabilities leading to raw data leakage 3) Maintain efficient and secure access management	1) Navigate through raw data without seeing it. 2) Prevent cyberattacks, conform with IRB requirements. 3) The app must integrate with OSU identity management.	1) Iterative refinement of cohorts promotes data understanding 2) Data processing is isolated and only aggregate-level outputs are returned to investigators. 3) Integrated with Shibboleth SP + manual access management.
Scalability	1) Handle hundreds of millions of medical records efficiently on different hosts. 2) Architecture can be scaled efficiently to incorporate multiple sites. 3) Manage a large number (hundreds or thousands) of patient cohorts at once	1) Single-machine servers may run out of memory if data is managed inefficiently. 2) Multi-site data would likely need multiple layers of aggregation and privacy protection. 3) The IDs representing each cohort would be megabytes in size, potentially causing storage problems.	1) Internal cohort representation is only linked to identifiers, not actual data. 2) Software components are isolated and can easily be moved/stacked across machines. 3) User cohorts are stored as dynamic views, essentially eliminating storage overhead for cohorts.

**Table 4:** Summary of User-Experience Objectives in Developing PCORsearch (Witte et al., 2023).

Objective	Design Goals	Obstacles	Results
Freedom of Analysis	1) Provide flexible cohort-definition capabilities. 2) Supply broadly applicable statistics and plots.	1) Letting users write raw SQL is confusing and a security risk. 2) Letting users write raw R is confusing and a security risk.	1) Create a simple, SQL-like grammar made specifically for PCORnet databases. 2) Automate plot generation. Users just press “SHOW RESULT”.
Ease Of Use (Sartipi and Dehmoobad, 2008)	1) Let users define a cohort with minimal training. 2) Give users a simple interface to work with terminology codes. 3) Allow sharing of cohorts between users.	1) The simplified grammar is not trivial to understand. 2) These terminology codes are heterogeneous with PCORnet and each other, complicating analysis. 3) Users do not have raw data access.	1) Allow users to specify terms of interest with button presses. 2) Create algorithms to map terminology codes to PCORnet and a GUI to explore different code taxonomies. 3) The profile page has an area to find recently defined cohorts.

**Table 5:** Mean Single-Query Latency of PCORsearch Cohort Discovery Query.

Query Type	Mean Latency (s) ± SD
Low Complexity, no LAB_RESULT_CM	6.890 ± 2.709
High Complexity, no LAB_RESULT_CM	35.04 ± 3.898
Low Complexity, with LAB_RESULT_CM	41.66 ± 2.911
High Complexity, with LAB_RESULT_CM	73.23 ± 3.381

**Table 6:** Summary of Regulatory Processes to Create PCORsearch, based on OSUMC.

Regulatory Process Completed	Regulatory Requirement Addressed
Acquire SQL server or modify existing server to accommodate access management and logging	Required for the web app itself to work
Get regulators certified to manage data as an honest broker	Required for PHI security compliance
Acquire IRB for devs to view PHI on SQL**	Required for PHI security compliance
Acquire SSL Certificate*	Required for the externally facing web app
Acquire DNS registration*	Required for the externally facing web app
Register with institutional service provider	Required for the externally facing web app
Modify or formalize data use agreement	Required for PHI security compliance
Perform internal risk assessment	Required for PHI security compliance
Perform IT security audit*	Required for the externally facing web app
Acquire application server*	Required for the externally facing web app
Modify institutional firewall for server to allow outgoing traffic on reverse proxy port*	Required for the externally facing web app

* Not required if using a locally hosted deployment.

** Recommended but not required.

**Table 7:** PCORsearch Data Model.

Table	Unique Identifier	Additional Identifiers
DEMOGRAPHIC	PATID	n/a
ENCOUNTER	ENCOUNTERID	PATID
DIAGNOSIS	DIAGNOSISID	PATID, ENCOUNTERID
DEATH	PATID	n/a
DEATH_CAUSE	PATID	n/a
CONDITION	CONDITIONID	PATID, ENCOUNTERID
VITAL	VITALID	PATID, ENCOUNTERID
PROCEDURES	PROCEDURESID	PATID, ENCOUNTERID
PRO_CM	PRO_CM_ID	PATID, ENCOUNTERID
PRESCRIBING	PRESCRIBINGID	PATID, ENCOUNTERID
OBS_CLIN	OBSCLINID	PATID, ENCOUNTERID
OBS_GEN	OBSGENID	PATID, ENCOUNTERID
LAB_RESULT_CM	LAB_RESULT_CM_ID	PATID, ENCOUNTERID

## Appendix A. Supporting information

Supplementary data associated with this article can be found in the online version at doi:10.1016/j.compbiolchem.2026.109200.
