# Supplementary material for: PCORsearch: A scalable, user-centric platform for self-service cohort discovery and feasibility analysis of PCORnet data
Source: Comput Biol Chem. Author manuscript; Available in PMC 2026 Aug 17. (PMC13479316; doi:10.1016/j.compbiolchem.2026.109200)
Supplement: Supplementary file 1 [file NIHMS2191251-supplement-Supplementary_Materials_3.docx]

Supplementary Material 3 details the tech stack used in PCORsearch. Certain items, like conda environments used in the app, can be found on the PCORsearch GitHub page. Please read below for details regarding platform tools and versioning.

End-User Layer:

Languages – HTML/CSS/JavaScript, Python, ASP.NET.

Frameworks – Plotly Dash, a React-based Python web framework. Conda is used, with a full environment provided on GitHub as a .yml file. .NET is used for the identity verification endpoint.

Application Layer:

Languages – Python, R

Frameworks – Flask

Data Layer:

Languages – SQL

Frameworks – SQL Server Management Studio

Infrastructure Layer:

Host – On-prem deployment, 32GB RAM 1TB Disk host machine, 24GB Shared SQL server.

Security – Shibboleth Service Provider

Networking – Microsoft Internet Information Services Manager, used as a reverse proxy.
